# SARS-CoV-2 Incubation Period during Omicron BA.5–Dominant Period, Japan

**DOI:** 10.3201/eid3001.230208

**Published:** 2024-01

**Authors:** Hao-Yuan Cheng, Andrei R. Akhmetzhanov, Jonathan Dushoff

**Affiliations:** Taiwan Centers for Diseases Control, Taipei, Taiwan (H.-Y. Cheng);; National Taiwan University, Taipei (A.R. Akhmetzhanov);; McMaster University, Hamilton, Ontario, Canada (J. Dushoff)

**Keywords:** COVID-19, respiratory infections, severe acute respiratory syndrome coronavirus 2, SARS-CoV-2, Omicron, incubation period, SARS, coronavirus disease, zoonoses, viruses, coronavirus, Japan

**To the Editor:** Ogata and Tanaka (*1*) estimated the mean incubation period was 2.9 (95% CI 2.6–3.2) days for SARS-CoV-2 strain Omicron BA.1 and 2.6 (95% CI 2.5–2.8) days for Omicron BA.5 during the Omicron-dominant period in Japan. Their earlier study reported a similar mean incubation period of 3.1 days for BA.1 (*2*). Their findings were derived from data collected through contact tracing efforts in Ibaraki Prefecture, Japan, which provided high accuracy in determining exposure time windows.

A potential concern is that their study only included cases that had a single exposure event and a 1-day exposure window. Although this concern was recognized by the authors as a study limitation, we emphasize that those criteria might bias results downward, especially when the disease is widespread. Persons that had longer incubation periods might have more opportunity for contacts or multiple exposure dates; thus, those with shorter incubation periods would be favored for inclusion. A more flexible case-selection approach might reduce bias, even though this approach would require methods to address uncertainty in actual infection timing.

In Taiwan, we collected data from the first 100 local symptomatic cases during the BA.1–dominant period (December 25, 2021–January 18, 2022), which were characterized by intensive case finding and contact tracing (A. Akhmetzhanov et al., unpub. data, https://doi.org/10.1101/2023.07.20.23292983). Among 69 cases with an identified exposure, only 4 had a 1-day exposure window. Using more comprehensive exposure windows, the estimated mean incubation period in Taiwan was 3.5 (95% CI 3.1–4.0) days, longer than Tanaka et al.’s estimates (*1*,*2*) but similar to estimates of 3.5 days from Italy (data collected during January 2022) (*3*) and South Korea (data collected during November–December 2021) (*4*) and estimates from a systematic review (3.6 days) (*5*). The estimates from Japan (*2*) appear to be the shortest periods reported across previously reviewed studies (*5*).
